# Hemodynamic Alterations Associated with Varying Aneurysm Sizes in the Aortic Arch

**DOI:** 10.3390/bioengineering13050519

**Published:** 2026-04-29

**Authors:** A B M Nazmus Salehin Nahid, Mashrur Muntasir Nuhash, Ruihang Zhang

**Affiliations:** Department of Mechanical and Industrial Engineering, University of Minnesota Duluth, Duluth, MN 55812, USA; nahid005@d.umn.edu (A.B.M.N.S.N.); nuhas001@d.umn.edu (M.M.N.)

**Keywords:** aortic arch aneurysm, aneurysm size, computational fluid dynamics (CFD), hemodynamics, patient-specific

## Abstract

Aortic arch aneurysms are uncommon but clinically significant due to their rapid growth and increasing rupture risk. Analyzing flow changes associated with aneurysm enlargement is essential for understanding mechanisms of disease progression. However, computational studies focusing on the aortic arch aneurysm remain limited. In this study, computational fluid dynamics (CFD) simulations were conducted under pulsatile flow conditions to investigate flow characteristics across different aneurysm sizes. A patient-specific aortic geometry was reconstructed and modified to generate three idealized aneurysm models with diameters of 45, 55, and 65 mm, along with a healthy reference model. Key hemodynamic parameters, including velocity distribution, flow recirculation, wall shear stress (WSS), oscillatory shear index (OSI) and helicity, were analyzed. The results demonstrated that increasing aneurysm size significantly disrupts normal flow patterns, leading to reduced flow velocities and progressively enhanced recirculation zones, particularly during the deceleration phase of the cardiac cycle. Enlarged aneurysms also exhibited consistently low WSS, elevated OSI, and disrupted helical flow patterns along the vessel walls. These adverse hemodynamic conditions are associated with intraluminal thrombus (ILT) formation, localized wall thinning, and increased risk of dissection or rupture. Overall, this study highlights the critical role of aneurysm size in shaping aortic arch hemodynamics and provides a computational framework for assessing disease progression and rupture potential.

## 1. Introduction

An aneurysm occurs due to the weakening and subsequent enlarging or dilation of blood vessel walls. Pathologically, a segment of the aorta is considered aneurysmal when its diameter exceeds 1.5 times the expected normal size [1]. While these lesions can develop anywhere along the vasculature, thoracic aortic aneurysms (TAA) present a particularly high clinical risk. It can lead to life threatening complications such as aortic dissections or rupture if left untreated. Aortic dissection is characterized by a tear in the innermost layer of the aorta wall, called intimal tear, allowing blood to flow between the layers of the aortic wall and create a “false lumen”. This limits blood delivery to vital organs and results in severe complications [2]. It severely weakens the aorta wall and can propagate upstream or downstream. A weakened aorta wall can lead to fatal rupture events. Particularly, aortic arch rupture has a mortality rate between 94% and 100% and these conditions often remain asymptomatic until the point of rupture [3,4]. Thus, early detection and accurate risk stratification are essential.

The thoracic aorta consists of four segments: the aortic root, the ascending aorta, the aortic arch, and the descending aorta. Among them, aortic arch aneurysms specifically account for approximately 21.3% of TAA cases [5]. It is a rare condition, but it enlarges faster and carries a higher risk of rupture than other aneurysms. For untreated patients, the actuarial five-year survival rate is only around 13%, with most deaths attributed to aortic rupture [6]. Currently, clinical imaging is currently the primary method for determining risk factors for aneurysm. This is generally assessed by tracking disease progression, such as absolute aneurysm diameter [7], aneurysm growth rate, along with presence of other physiological conditions like hypertension, or genetic conditions such as Marfan syndrome [8]. However, relying solely on anatomical risk assessment may fail to identify high risk patients who suffer complications below standard size thresholds [9]. To address these limitations, computational fluid dynamics (CFD) has emerged as a very important tool to complement risk assessment by analyzing the hemodynamics of blood flow and its effect on the vessel wall [10,11].

CFD has been extensively utilized to investigate the role of hemodynamics in aneurysm progression across various arterial locations. Castro et al. analyzed 26 cases in anterior communicating arteries reconstructed from 3D angiography. Their results showed that aneurysms with higher peak WSS were more likely to rupture and suggested that focused flow jets or impingement patterns may play an important role in rupture [12]. Similarly, Xiang et al. examined 119 intracranial aneurysms, comparing hemodynamic characteristics between 38 ruptured and 81 unruptured cases using patient-specific CFD models derived from 3D angiograph [13]. Their results indicated that most rupture cases had complex and multiple vortex flow, while unruptured cases had simpler, single-vortex patterns. In addition, ruptured aneurysms exhibited lower normalized WSS and higher OSI. Cebral et al. conducted a study involving 128 patients with intracranial aneurysm and found that elevated peak wall stress was related to rupture [14]. In a longitudinal study, Takao er al. followed 100 medium size unruptured aneurysms, 13 of which later ruptured during the follow-up period, and reported significantly lower minimum time averaged WSS in the ruptured group [15]. Beyond intracranial aneurysms, similar hemodynamic trends have been observed in abdominal aortic aneurysms (AAA). Bappo et al., in a cohort of 295 patients, identified that low WSS as an independent factor associated with both aneurysm expansion rate and rupture risk [16]. Consistently, based on a seven-patient AAA cohort, Boyd et al. reported that rupture is often associated with regions of low WSS, a finding supported by Parker et al., who observed that ruptures in the common iliac artery often occur at sites by low shear stress [17,18]. In the context of the aortic arch, Numata et al. conducted a study on aortic arches of six different patterns based on patient-specific CT scan images [19]. They found that dilated aorta causes turbulent flow patterns in the aortic arch. They further suggested a possible correlation between elevated OSI and the initiation of acute aortic dissection. Overall, these findings indicate that dilation of the aortic arch can significantly alter local hemodynamics, potentially contributing to pathological progression through increased flow disturbance and adverse shear environments.

Despite the recognized importance of hemodynamic factors, systematic investigations into how progressive enlargement influences blood flow pattern and hemodynamic behavior within the complex geometry of the aortic arch remain relatively limited. In this study, CFD simulations were conducted using a patient-specific aortic arch model to address this gap. A healthy geometry was modified to represent three idealized aneurysm sizes (45 mm, 55 mm, and 65 mm), enabling a comparative analysis of key hemodynamic parameters, including velocity streamlines, WSS, OSI and helicity. The primary objective is to analyze how increasing aneurysm size alters normal flow patterns and mechanics in the aorta. These findings aim to provide insights that may support improved clinical assessment of rupture risk in aortic arch aneurysms.

## 2. Methods

### 2.1. Model Configuration and Boundary Conditions

A patient-specific healthy aorta was obtained from a publicly available database named K6.nrrd [20]. It was then reconstructed in SolidWorks—version 2024 (Dassault Systèmes SolidWorks Corp., Waltham, MA, USA) to closely replicate the patient-specific anatomy. For this purpose, the file was first converted to a mesh file in STL (stereolithography) format using 3D slicer (version 5.8.1, slicer.org). Since SolidWorks works better with mesh face size less than 20,000, additional remeshing was carried out then. The quadric edge collapse decimation method in open-source MeshLab—version 2023.12 (meshlab.net) was utilized to keep the number of faces under target while retaining the original shape of the geometry.

**Figure 1:**
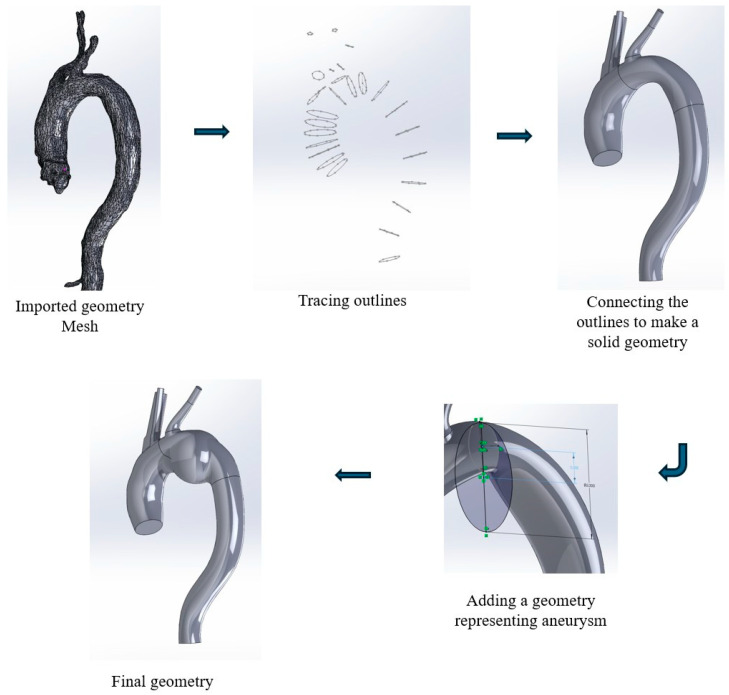
Creating a geometry with aneurysm from imported mesh in SolidWorks. The arrows in the figure indicate the general model creating steps.

**Figure 2:**
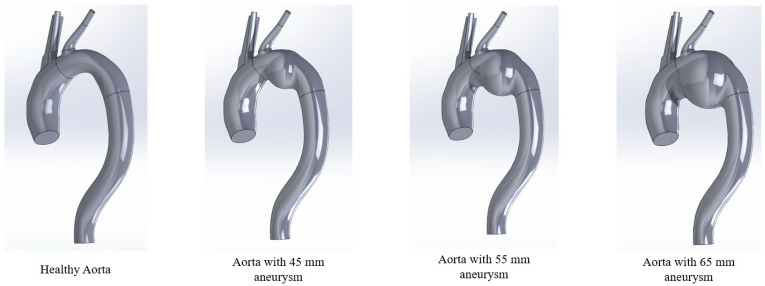
Aorta at different stages of aneurysm.

Figure 1 shows the detailed steps to create a patient-specific geometry with aneurysm from a mesh file in SolidWorks. First, the geometry mesh file needs to be imported as a surface body. Then, the periphery of the aorta was captured by a closed curve exploiting the 3D sketch on plane and spline feature. The spline feature enables capture of irregularities and non-uniform features of the aorta. A series of closed curves were drawn following the path of the aorta to maintain the shape of the aorta. After that, a 3D body was created by connecting the closed curves using the lofted boss/base feature. The brachiocephalic trunk, the left common carotid, and the left subclavian artery was simplified to reduce the complexity of the aorta. Finally, a sphere was added to the geometry of the arch to represent aneurysm. The diameter of the sphere was adjusted to represent different sizes of aneurysm. Three configurations were modified with the following aneurysm sizes (by diameter size): 45 mm, 55 mm, and 65 mm, respectively, as shown in Figure 2. The aneurysm diameters were selected in this study to reflect the clinical progression of the disease and standard surgical guidelines. A diameter of 55 mm corresponds to the established clinical threshold for surgical intervention in asymptomatic patients according to the 2022 ACC/AHA and ESVS guidelines [21,22]. The 45 mm and 65 mm cases were included to represent moderate and high-risk conditions, respectively, as rupture risk increases markedly beyond 60 mm [23,24]. This range enables a systematic assessment of hemodynamic changes across clinically relevant aneurysmal stages.

In addition, this hybrid modeling approach balances patient-specific anatomy with controlled parametric variation in this study. Although the aneurysm sac is idealized as a sphere to isolate the effect of diameter size, anatomical realism is preserved by maintaining the patient’s original 3D arch curvature, aortic centerline, and the exact spatial distribution of the supra-aortic branches.

**Figure 3:**
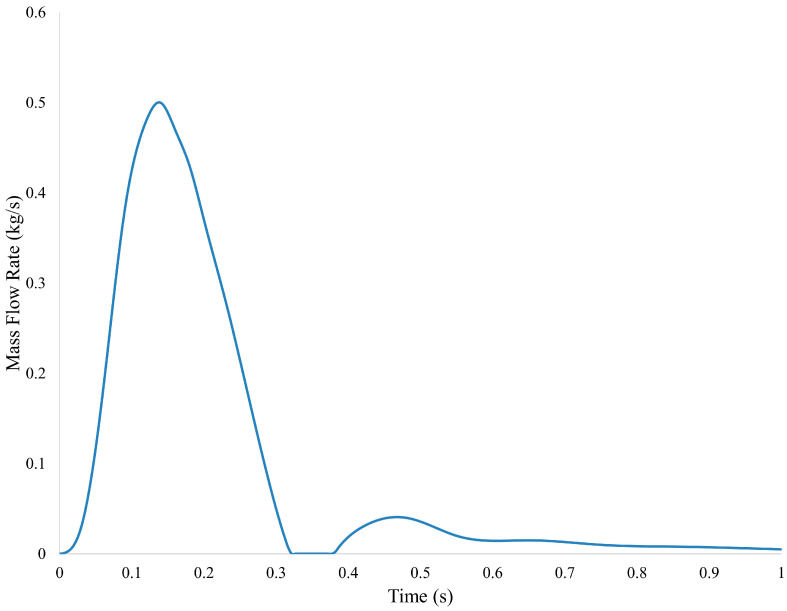
Mass flow rate waveform boundary condition for the inlet [25].

In this numerical study, CFD simulations have been performed for these various aneurysm conditions using Ansys FLUENT 2023 R2 (Ansys, Canonsburg, PA, USA) under the pulsatile flow condition. As shown in Figure 3, a pulsatile waveform of mass flow rate was applied at the inlet of the simulation [25]. Outlets were set as outflow conditions, with the main outlet set with 85% of the flow, and the three secondary outlets with 5% of the flow. The aortic pressure was set at 120 mmHg. The aorta walls were assumed rigid with the no-slip boundary condition. In addition, blood was modeled as the Newtonian fluid with a constant dynamic viscosity of 0.0035 Pa·s and a density of 1060 kg·m^−3^. This assumption is justified for large arteries such as the aorta, where high shear rates minimize non-Newtonian effects, resulting in negligible differences in key hemodynamic parameters compared to non-Newtonian models [24,26].

### 2.2. Governing Equations

The Continuity equation and Navier–Stokes equation were used to calculate the flow in all cases [27]:

(1) $$\frac{\partial \left(\rho u_{i}\right)}{\partial x_{i}}=0$$

(2) $$\frac{\partial }{\partial x_{j}}\left(\rho u_{i}u_{j}\right)=-\frac{\partial p}{\partial x_{i}}+\frac{\partial }{\partial x_{j}}\left\{\mu \left[\frac{\partial u_{i}}{\partial x_{j}}-\bar{\rho u_{i}^{\prime }u_{j}^{\prime }}\right]\right\}$$

Here, ρ is the density of the fluid in Kg·m^−3^, u represents the fluid velocity (m·s^−1^), p indicates pressure (Pa), µ is the fluid dynamic viscosity (Pa·s). The Reynolds term, $-\bar{\rho u_{i}^{\prime }u_{j}^{\prime }}$, can be further calculated through the Boussinesq hypothesis:

(3) $$-\bar{\rho u_{i}^{\prime }u_{j}^{\prime }}=\mu_{t}\left[\frac{\partial u_{i}}{\partial x_{j}}+\frac{\partial u_{j}}{\partial x_{i}}\right]-\frac{2}{3}\rho k\delta_{ij}-\frac{2}{3}\mu_{t}\frac{\partial u_{k}}{\partial x_{k}}\delta_{ij}$$

In Equation (3), $\delta_{ij}$ represents the Kronecker delta, k is the turbulent kinetic energy (TKE) (m^2^·s^−2^), and µt is turbulent viscosity (Pa·s). The k-ω SST turbulence model was utilized in this study, where k represents TKE and ω indicates the diffusion of turbulent energy. This turbulence model employs the k-ω model for near wall accuracy and switches to the k-ε model away from the wall for improved stability and accuracy in complex flows in presence of turbulence.

### 2.3. Mesh Sensitivity Analysis

To reliably capture wall properties of the aorta such as WSS via numerical solutions, the flow close to the wall must be accurate. For this purpose, the first layer of the fluid mesh needs to be sufficiently close to the wall. This distance is represented by a dimensionless distance $y^{+}$, which should be less than 1 to achieve correct wall properties. It is calculated by the formula:

(4) $$y^{+}=\frac{T_{1}u_{t}}{\mu /\rho }$$

(5) $$u_{t}=\sqrt{\frac{\tau_{max}}{\rho }}$$

**Figure 4:**
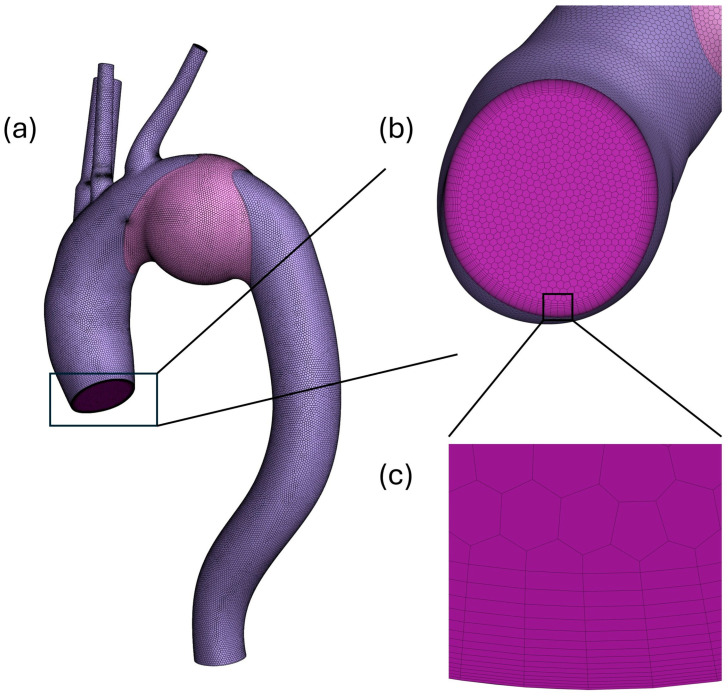
Mesh of the aorta: (**a**) example of a mesh of the entire aorta, (**b**) mesh at the inlet of the aorta, (**c**) inflation layers at the inlet.

Here, $\tau_{max}$ is the maximum wall shear stress during a cardiac cycle. To achieve a desired $y^{+}$, of less than 1, an inflation layer consisting of 15 boundary layers was introduced to the fluid mesh. Smooth transition method was used to determine the heights of the boundary layers, with a transition ration of 0.272 and a growth rate of 1.15. In this method, the height of the cell was lowest close to the wall, gradually increasing as the mesh moves towards the center of the flow and finally merges with the main mesh body. Figure 4 shows the generated mesh of the aorta.

The mesh was generated in Ansys Fluent 2023 R2 with fluent meshing. To optimize the mesh density, a mesh sensitive analysis was performed on the 55 mm aneurysm model. Four different mesh sizes, ranging from 0.125 million cells to 1.91 million cells were utilized. The average flow velocity at peak systole was compared across these meshes, shown in Table 1. The change for average velocity at 1.91 million cells was less than 3 percent compared to 1 million cell count. Thus, the mesh with 1 million cells was selected for the final simulation to balance between numerical accuracy and computational efficiency. The minimal variation between the medium and fine meshes indicates that the solution is both mesh-independent and numerically stable.

Additionally, Table 2 shows optimization of timesteps size. Like mesh analysis, average velocity was analyzed at peak systole on the 55 mm aneurysm model. The optimized mesh size of 1 million cell count was utilized, based on the mesh sensitive analysis. From the table, timesteps size of 0.005 s and 0.0025 s shows very small change in performance. Consequently, 0.005 s was selected as the optimized timestep. This analysis confirms that the selected temporal resolution accurately captures the pulsatile flow features without numerical instability. Together, both the verification steps indicate that the reported hemodynamic patterns are a result of the physical model rather than numerical errors.

### 2.4. Quantities of Interest

To systematically observe and describe the hemodynamics properties within the aneurysm models, we analyzed the key flow variables derived from the numerical simulations. These selected variables were used to capture the fundamental features of both intra-aneurysmal flow structures and wall-flow interactions within the cardiac cycle.

- **Wall shear stress (WSS):** WSS is the tangential frictional force generated by flowing blood along the luminal endothelial surface. It is defined as the force per unit area.
  (6) $$WSS=\mu {\left(\frac{\partial u}{\partial y}\right)}_{y=0}$$
  Here, μ is the dynamic viscosity, u is the blood velocity, and y indicates distance from the artery wall.
- **Oscillatory shear index (OSI):** A dimensionless scalar number that characterizes the areas subjected to large directional variation in WSS during a cardiac cycle. It is calculated by the following equation:
  (7) $$OSI=\frac{1}{2}\left(1-\frac{\left|\int_{0}^{T}WSSdt\right|}{\int_{0}^{T}|WSS|dt}\right)$$
  Notably, OSI ranges from 0 to 0.5. A value of 0 indicates the instantaneous WSS vector at a specific time aligns well with the time averaged WSS vector throughout the cardiac cycle, reflecting the absence of directional oscillation. On the other hand, a value of 0.5 shows extremely oscillatory shear behavior.
- **Absolute normalized helicity:** A directionally independent, dimensionless metric used to quantify the strength of helical flow. Its value ranges from 0 to 1, where 0 indicates the absence of helical flow, and 1 corresponds to maximally helical flow.
  (8) $$\mathrm{Absolute}\;\mathrm{normalized}\;\mathrm{helicity}=\left|\frac{V\cdot \omega }{\left|V\right|\left|\omega \right|}\right|$$
  In this equation, V represents the velocity vector and ω is the vorticity vector.
- **Pressure loss coefficient (K):** it quantifies the energy loss associated with flow separation, turbulence, and geometry changes. This dimensionless parameter can be calculated using the following equation:
  (9) $$\mathrm{Pressure}\;\mathrm{loss}\;\mathrm{coefficient}=\frac{∆P}{\frac{1}{2}\rho U^{2}}$$
  where ΔP is the pressure drop (Pa) across the aorta, ρ is the density of the fluid in Kg·m^−3^, and U represents the velocity in m·s^−1^.

## 3. Results

### 3.1. Velocity Profile and Flow Structure

**Figure 5:**
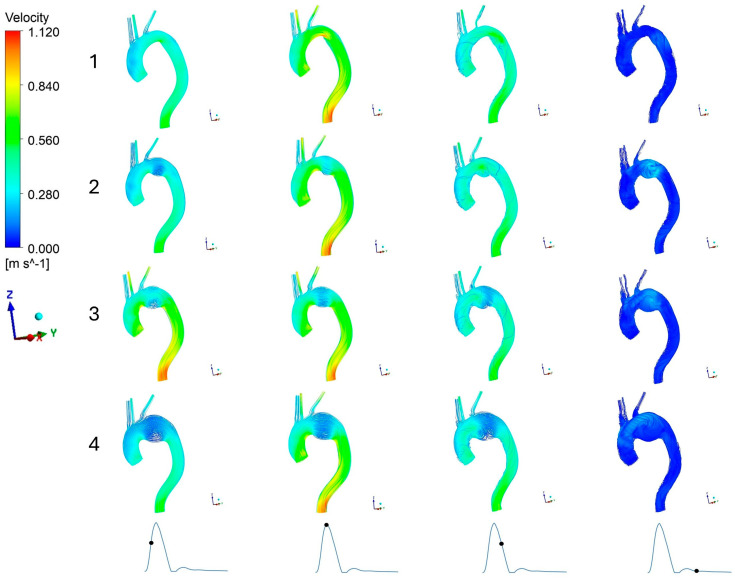
Velocity streamline in aorta: 1. No aneurysm, 2. 45 mm aneurysm, 3. 55 mm aneurysm, 4. 65 mm aneurysm. The black dot on the inlet waveform on the bottom of this figure represents the time of the cardiac cycle at which velocity was calculated: (from left to right) acceleration, peak systole, deceleration, and diastole, respectively.

Figure 5 illustrates the velocity streamline across the aorta for various aneurysm models of the cardiac cycle: acceleration, peak systole, deceleration, and diastole, denoted by black dot on the inlet waveform. The different aneurysm sizes are marked with numbers one to four; with number one representing the healthy aorta as the reference model and numbers two to four representing 45 mm, 55 mm, and 65 mm aneurysm size, respectively.

Comparing the velocity streamlines, a clear trend can be observed: as the aneurysm expands, the localized flow velocity within the aneurysm sac noticeably decreases. This flow reduction is most prominent in the 65 mm model, which also develops notable presence of the recirculation zone. Additionally, the formation of these disturbed flow structures is highly dependent on the cardiac phase. The acceleration and peak systole phases show no significant recirculation. However, at deceleration stage, distinct recirculation begins to emerge in the aneurysmal region. These swirling flow patterns get more pronounced with increase in the overall aneurysm size as shown in Figure 5.

**Figure 6:**
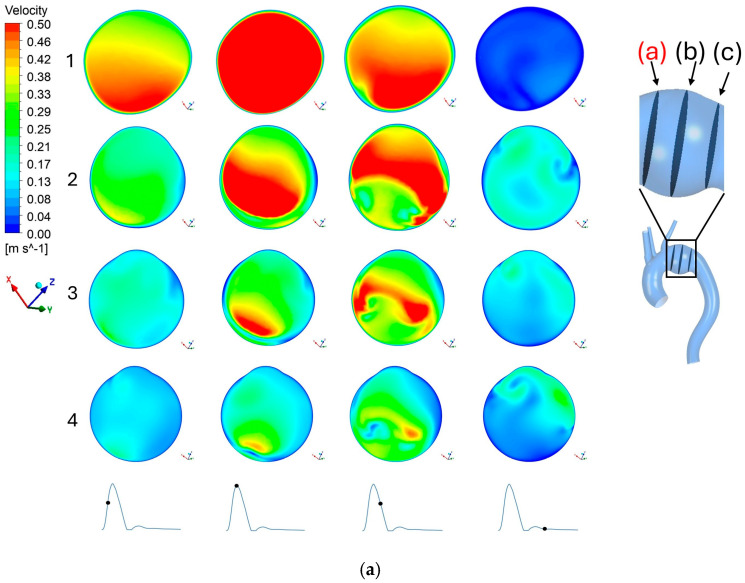

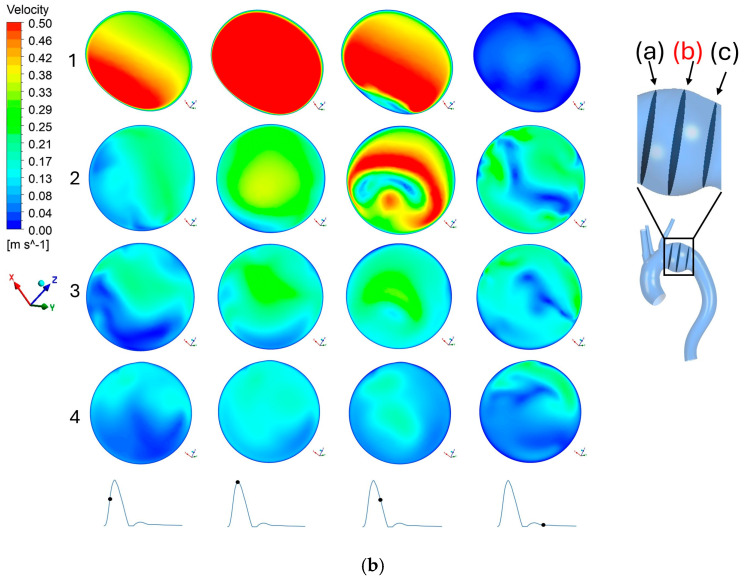

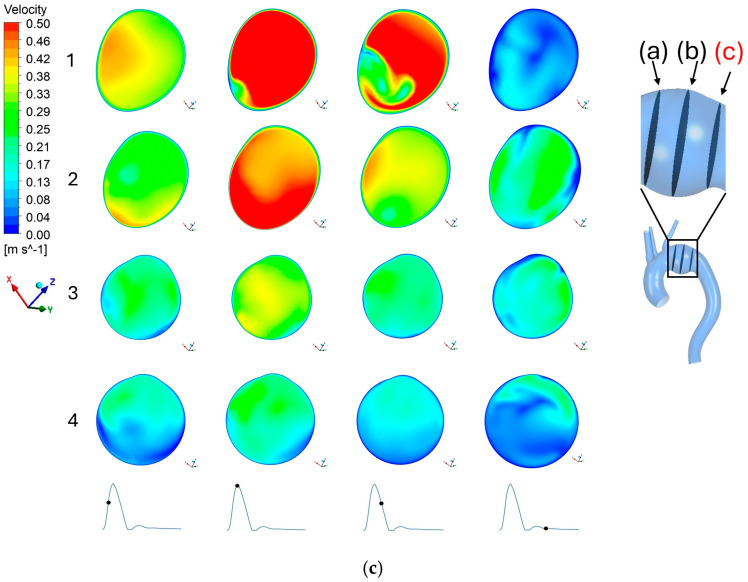
Velocity profile on plane (**a**): 1–4 represents healthy, 45 mm, 55 mm, and 65 mm aneurysm size, respectively. Velocity profile on plane (**b**): 1–4 represents healthy, 45 mm, 55 mm, and 65 mm aneurysm size, respectively. Velocity profile on plane (**c**): 1–4 represents healthy, 45 mm, 55 mm, and 65 mm aneurysm size, respectively. The black dot on the inlet waveform on the bottom of each figure represents the time of the cardiac cycle at which velocity was calculated: (from left to right) acceleration, peak systole, deceleration, and diastole, respectively.

To examine the internal flow dynamics more closely, Figure 6 presents the cross-sectional velocity across three different planes to analyze blood flow within the aneurysm: the entry plane of aneurysm (Plane a), the mid plane (Plane b), and the exit plane (Plane c). These cross sections complement the streamline analysis and further support that velocity decreases with increase in aneurysm size.

At the entry plane (Figure 6a), flow velocity in the healthy aorta is uniform, showing the highest velocity magnitude of all the tested conditions. However, as the aneurysm size increases, the uniform velocity profile rapidly breaks down. The overall velocity drops significantly, and the flow distribution becomes increasingly uneven across the cross-section. This irregular velocity distribution is an important observation, as it likely induces the disturbed recirculation zones that develop further downstream. This phenomenon can be confirmed in the following planes (Figure 6b,c), showing that the slower, disrupted flow persists through the mid-point and exit of the aneurysm. Ultimately, these cross-sectional views strongly support the streamline observation, confirming that larger aneurysms inherently reduce localized blood velocity and disturb normal flow patterns.

### 3.2. Wall Shear Stress (WSS)

**Figure 7:**
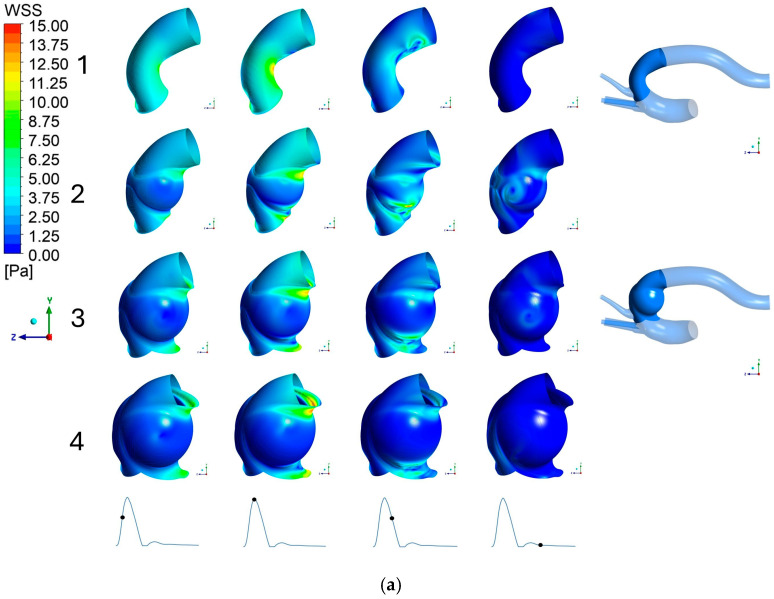

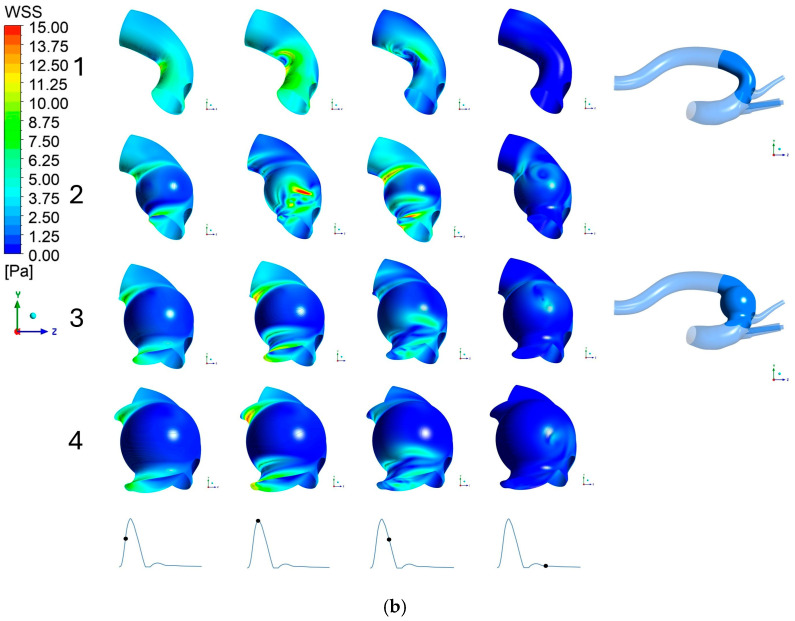
(**a**) Wall shear stress at aneurysm wall, YZ+ plane in aorta: 1. No aneurysm, 2. 45 mm aneurysm, 3. 55 mm aneurysm, 4. 65 mm aneurysm. (**b**) Wall shear stress at aneurysm wall, YZ− plane in aorta: 1. No aneurysm, 2. 45 mm aneurysm, 3. 55 mm aneurysm, 4. 65 mm aneurysm. The black dot on the inlet waveform on the bottom of each figure represents the time of the cardiac cycle at which velocity was calculated: (from left to right) acceleration, peak systole, deceleration, and diastole, respectively.

Figure 7a,b shows the distribution of wall shear stress (WSS) across the specific region defined at the aneurysm wall, viewed from both the YZ+ and YZ− planes. The numbers one to four in Figure 7 represent healthy aorta reference model and modified aorta with 45 mm, 55 mm, and 65 mm aneurysm, respectively. To clarify the analyzed region, the right side of the figure of two aorta models highlights the targeted aneurysm boundary in blue on both a healthy reference model and the 55 mm aneurysm configuration as example, while the remaining vessel is rendered transparent.

In the healthy aorta (Model 1), the WSS distribution is relatively consistent and uniform across the equivalent wall region throughout the cardiac cycle. The only notable observation is the exception along the inner curvature of the aortic bend, where higher stress concentrations are observed, becoming particularly evident as flow approaches peak systole. However, as the aneurysm size expands, the mechanical forces exerted on the vessel wall shift dramatically. The overall WSS across the aneurysmal sac is significantly reduced in the 45 mm aneurysm model compared to the healthy baseline, except for a localized concentration of WSS on the YZ− plane during peak systole.

Moreover, this reduction phenomenon in shear stress becomes even more pronounced and widespread as the aneurysm size continues to expand. Particularly, both the 55 mm and 65 mm models display low WSS across nearly the entire surface of the aneurysm wall. Interestingly, higher WSS was observed at the geometrical junction where the aneurysm meets the native aorta. Rather than reflecting a real physiological phenomenon, this localized stress concentration likely arises as an artifact of the simplified transition in the idealized CAD geometry.

### 3.3. Oscillatory Shear Index (OSI)

**Figure 8:**
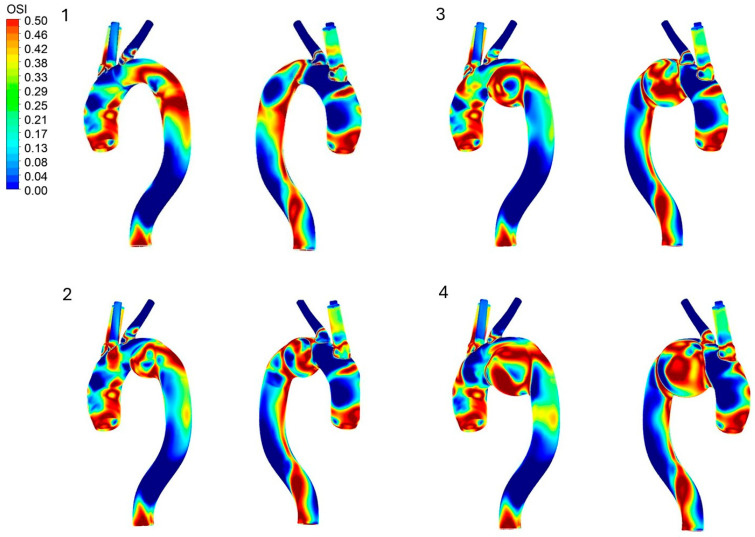
Oscillatory shear index at the aorta wall in the aorta: 1. No aneurysm, 2. 45 mm aneurysm, 3. 55 mm aneurysm, 4. 65 mm aneurysm.

Figure 8 shows the distribution of the oscillatory shear index (OSI) across the aorta wall for all four configurations. In the healthy aorta, high OSI can be observed at the inlet region and localized sections of the descending aorta. Although the OSI near the inlet remains relatively consistent under all tested conditions, aneurysm formation and growth produce pronounced changes in the downstream OSI pattern.

Notably, as the aneurysm size enlarges, the elevated OSI previously observed in the descending aorta diminishes, with the aneurysm wall emerging as the primary region of high shear oscillation. In the 55 mm and 65 mm aneurysm models, this effect is particularly pronounced, with extensive, highly concentrated regions of high OSI spanning the aneurysmal sac. Interestingly, instead of a simple monotonic increase in OSI intensity with diameter, the 55 mm and 65 mm configurations show a comparable distribution of shear oscillation.

These localized OSI peaks observed in the enlarged aneurysms correspond closely to the complex recirculating flow patterns and vortex formations established during the deceleration stage of the cardiac cycle. High OSI indicates a flow environment with consistently fluctuating shear directions, reflecting highly unstable hemodynamic conditions. When paired with the localized regions of low WSS, these elevated OSI zones indicate a hostile mechanical environment along the vessel wall. From a clinical perspective, this combination of low WSS and high OSI is strongly associated with adverse disease progression, playing a critical role in contributing to intraluminal thrombus (ILT) formation, localized wall degradation, and an increased risk of aortic dissection or rupture.

### 3.4. Absolute Normalized Helicity

**Figure 9:**
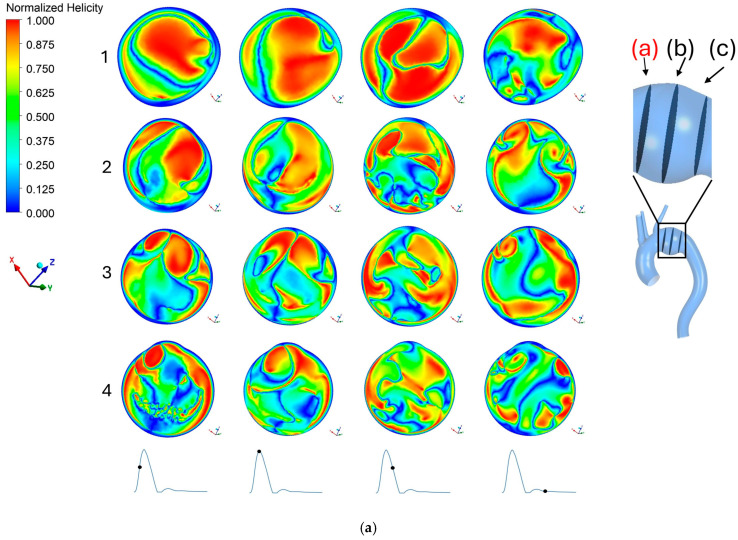

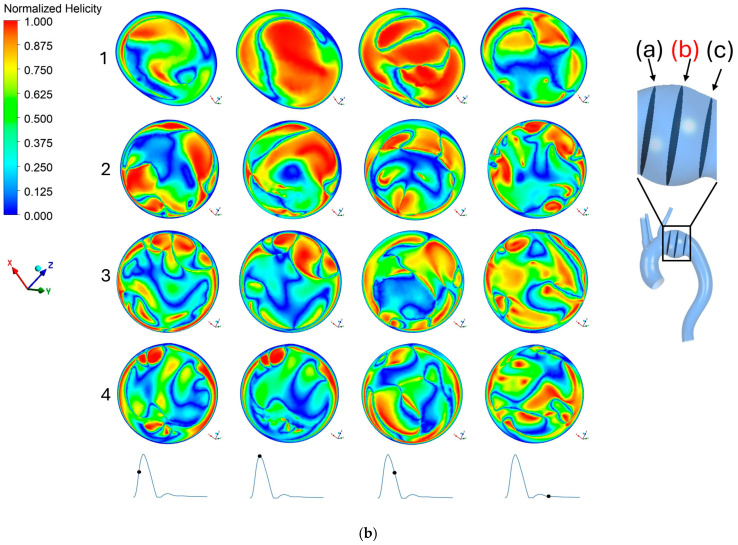

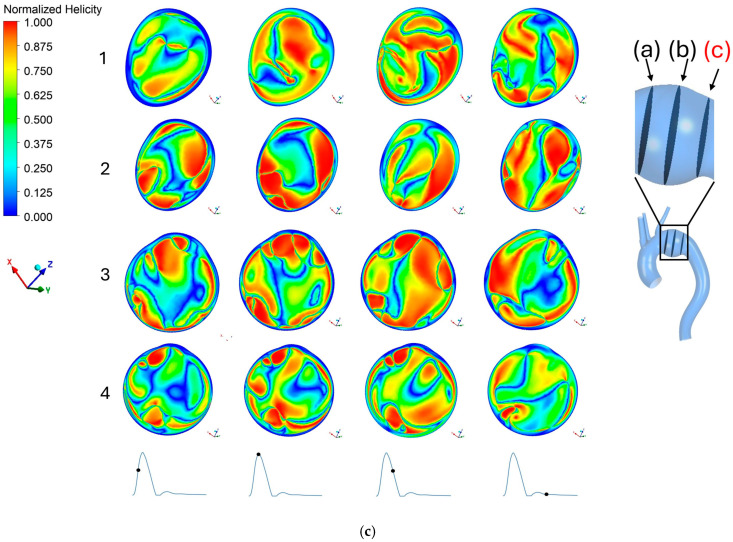
Absolute normalized helicity on plane (**a**) in aorta: 1. No aneurysm, 2. 45 mm aneurysm, 3. 55 mm aneurysm, 4. 65 mm aneurysm. Absolute normalized helicity on plane (**b**) in aorta: 1. No aneurysm, 2. 45 mm aneurysm, 3. 55 mm aneurysm, 4. 65 mm aneurysm. Absolute normalized helicity on plane (**c**) in aorta: 1. No aneurysm, 2. 45 mm aneurysm, 3. 55 mm aneurysm, 4. 65 mm aneurysm. The black dot on the inlet waveform on the bottom of each figure represents the time of the cardiac cycle at which velocity was calculated: (from left to right) acceleration, peak systole, deceleration, and diastole, respectively.

Figure 9a–c exhibits the distribution of absolute normalized helicity across three different cross-sectional planes within the aneurysm using a point cloud method. In cardiovascular fluid mechanics, helicity is a key parameter, describing the helical rotation of blood flow within the vasculature.

In the healthy reference model, the aorta reveals a strong, highly organized helical flow structure. As observed in Figure 9a, this high helicity is particularly prominent at the entry plane (Plane a) and is sustained throughout the acceleration, peak systole, and deceleration phases of the cardiac cycle. However, aneurysm enlargement substantially alters the native flow architecture. As the vessel expands, the forward-rotating momentum is disrupted by the sudden increase in volume, leading to the formation of stagnant recirculation zones. As a result, all three aneurysm models (45, 55, and 65 mm) display an uneven, scattered, and weakened distribution of helicity across the analyzed planes. This loss of organized helical flow further destabilizes hemodynamics, directly resulting in the chaotic flow patterns and high shear stresses observed within the enlarged aneurysm sacs.

### 3.5. Pressure Loss Coefficient (K)

**Figure 10:**
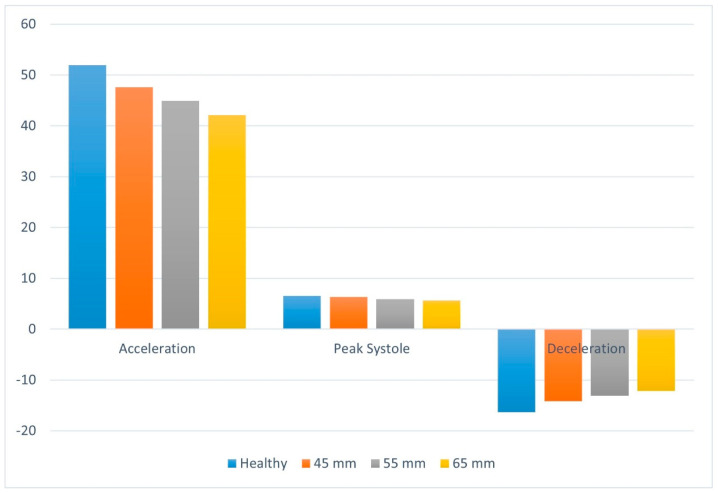
Comparison of pressure loss coefficient for different sizes of aneurysm for acceleration, peak systole, and deceleration.

The pressure loss coefficient (K) serves as a key parameter to evaluate the energy dissipation and flow resistance. As summarized in Table 3 and visually compared in Figure 10, the pressure loss coefficient values fluctuate significantly across the acceleration, peak systole, and deceleration stages, demonstrating a clear dependency on both the cardiac phase and the aneurysm size.

During the acceleration stage, the pressure loss coefficient reaches the highest value among all the three models. In the healthy aorta, K is 51.929. Interestingly, an inverse relationship is observed between the pressure loss coefficient and aneurysm enlargement during this phase. The K value consistently reduces to 47.58, 44.89, and 42.08 for the 45 mm, 55 mm, and 65 mm aneurysm sizes, respectively. This reduction indicates that the enlarged aneurysmal volume may locally reduce flow velocity and decrease the sharp pressure gradients typically associated with rapid flow acceleration. At peak systole, the pressure loss coefficients are slightly lower than during acceleration, decreasing modestly from 6.58 in the healthy aorta to 6.36, 5.9 and 5.6 in the 45 mm, 55 mm, and 65 mm models, respectively. Although differences are small, the consistent decline indicates that aortic dilation continuously alters energy loss even at maximum flow.

Conversely, the deceleration stage presents a distinct hemodynamic behavior characterized by negative K values, reflecting complex pressure recovery and flow reversal as the forward driving force diminishes. In the healthy aorta, the K value is highly negative at −16.32. As the aneurysm size increases, the K value increases (becoming less negative), registering at −14.19, −13.09, and −12.13 for the 45 mm, 55 mm, and 65 mm models, respectively. This progressive increase in K during deceleration is highly indicative of the increasing flow disturbances and stronger recirculation zones in larger aneurysms. As seen in the velocity streamlines, enlarged aneurysms trap circulating blood, dissipating energy in a chaotic manner and impeding normal pressure recovery during the deceleration.

## 4. Discussion

The current results indicate that flow velocity reduces as aneurysm size increases. Furthermore, the presence of recirculation zones becomes more pronounced with larger aneurysms, particularly during the deceleration stage. Boyd et al. previously correlated area of rupture with recirculating, low velocity blood flow [17]. They suggested that deposition of intraluminal thrombus (ILT) continuously occurs at these low velocity and low WSS sites [17]. Azrani et al. also found that low WSS was associated with ILT growth [28]. ILT is a complex structural compound composing platelets, blood proteins, cellular debris, and blood cells [29]. Within the aneurysmal environment, platelets that are activated in high-stress regions tend to accumulate in low-shear regions, thereby forming a thrombus [30]. It has been theorized that vortical structures formed within the aneurysm sac may play a critical role in the formation of thrombus. Additionally, the recirculating blood flow inside aneurysm may increase residence time, trapping cellular components and facilitating ILT progression [31,32]. Once formed, the ILT leads to complications on the aneurysm wall. It may aggravate wall inflammation and weakening by depriving the aneurysm wall of oxygen [33]. Moreover, as the thrombus matures, it can reduce aortic wall compliance and increase the risk of dissection. Consequently, accelerated ILT growth has been linked with increasing the risk of aneurysm rupture, where the failure of the thrombus itself can precipitate a fatal rupture event [34].

Historically, both abnormally high and distinctly low WSS have been implicated in the growth and rupture of aneurysm [13,14,15,35]. Meng et al. presented an article unifying these findings [36]. In their framework, aneurysm initiation is linked to high WSS near the jet impingement region. After initiation, aneurysm growth depends on the shear environment. Under high WSS, matrix breakdown and cell apoptosis may dominate, allowing rupture even in relatively small aneurysms [36]. Under low WSS, slow recirculation and disturbed flow can enhance inflammation and atherosclerotic plaque development, which can further amplify inflammatory damage and promote enlargement. They also argued that WSS may vary with geometrical changes in the aneurysm, and across different stages of aneurysm development [36]. Additionally, low WSS can be associated with thin aneurysmal walls, which is considered a risky condition for unruptured walls. Furthermore, diminished WSS is strongly associated with localized thinning of the aneurysmal wall—a highly precarious state for unruptured lesions. For instance, Kadasi et al. conducted CFD analysis on 16 patients with cerebral aneurysms and compared relationship between hemodynamic factors and localized thinning of aneurysm [37]. They suggested a correlation between low WSS and thinning of the aneurysm wall. Our simulations consistently indicate a pronounced drop in WSS corresponding to larger aneurysm volumes, aligning firmly with these established pathophysiological models. Furthermore, regarding systemic flow resistance, our data reveals that pressure loss coefficient decrease with the aneurysm size increases during the acceleration phase. This contrasts with previous studies, which reported an increase in the pressure loss coefficient with aneurysm size [38]. However, at peak systole, the coefficient shows only a slight decrease, whereas during flow deceleration, energy dissipation increases is observed alongside aneurysm dilation.

Additionally, the analysis of the OSI demonstrates that a higher concentration of OSI within the aneurysmal regions compared to the baseline healthy aorta. This elevated OSI is generally associated with low-velocity recirculating flow and reduced WSS, a combination that closely reflects the hemodynamic patterns observed in our models. Moreover, in the OSI simulation results, the comparable OSI levels in the 55 mm and 65 mm cases, rather than a strictly monotonic increase with size, suggest a hemodynamic threshold for this specific aortic arch geometry. As the aneurysm expands from 45 mm to 55 mm, the flow shifts from relatively organized streamlines to a dominant recirculating vortex. At 55 mm, the sac volume is sufficient to sustain this large-scale vortex during the deceleration phase. Further expansion to 65 mm mainly increases the area exposed to elevated OSI, without materially altering the flow structure or peak oscillation intensity, indicating a saturation state of the underlying hemodynamics. Notably, similar behavior has been widely reported in other vascular beds. Takehera et al. observed that dilated infrarenal aorta showed more vortex, lower WSS and elevated OSI compared to non-dilated aorta [39]. Moon et al. found that consistent correspondence of rapture sites with low WSS, recirculating flow and high OSI [40]. Additionally, in a study on six patient-specific models, Hohri et al. identified that high OSI concentration near the primary entry site of acute type A aortic dissections [41]. Elevated shear oscillation is therefore considered a key factor in both the initiation and progression of aneurysms [42]. While OSI serves as a key indicator, its evaluation alongside WSS and recirculation metrics provides a more comprehensive assessment of the adverse hemodynamic environment. Collectively, in this study, the observed increase in flow recirculation, reduction in WSS in aneurysm, and presence of elevated OSI with increase in aneurysm size indicate a progressively adverse hemodynamic environment. These conditions have been widely associated with ILT formation, wall degeneration, dissection, and rupture in prior related studies. It should be emphasized, however, that such pathological implications are inferred from the existing literature, as the present work is limited to computational hemodynamic analysis and does not directly model these biological processes.

While the literature specifically addressing aortic arch hemodynamics remains limited, our observations were frequently contextualized against data from abdominal and cerebral aneurysms. Although inherent differences in cellular composition and local physiology prevent a perfect one-to-one comparison, the underlying mechanics of vessel failure are broadly analogous across vascular beds. According to the principles of biomechanical failure, dissection or rupture occurs universally when wall shear or fluid pressure exceeds the maximum tensile strength of the local tissue [43]. Therefore, under similar deleterious hemodynamic loads, comparable pathogenic trends should be expected regardless of the anatomical site.

Despite the insights provided by this study, several limitations should be acknowledged. Primarily, the current study relied on the assumption of rigid wall boundary conditions for the aorta. This assumption can inherently limit the accuracy of derived wall-based parameters such as WSS. Future investigations would benefit from integrating fluid–structure interaction (FSI) models to accurately simulate aortic elasticity. Furthermore, the applied inlet mass flow waveform was not patient-specific, the outlet boundary conditions were constrained by fixed pressures, and the dynamic influence of the aortic root was omitted to simplify the inlet conditions in this study. Moreover, this present computational study was based on a single reconstructed patient-specific geometry. Thus, a large-scale study incorporating diverse patient datasets could provide a more comprehensive understanding of how hemodynamics changes with aneurysm size in the future. Lastly, although the complexity of secondary flows in the aortic arch makes experimental studies technically challenging, the present work provides an important computational framework for identifying the critical diameters that merit targeted experimental investigation. To address it, future work will involve fabricated patient-specific, 3D-printed phantom models based on the geometries examined in this study, followed by particle image velocimetry (PIV) measurements to provide quantitative validation of the present numerical results.

## 5. Conclusions

This study aimed to improve the understanding of hemodynamics within the aortic arch by analyzing the effects of varying aneurysm sizes. Using a patient-specific geometry modified to represent three different aneurysm sizes (45, 55, and 65 mm) and a healthy reference model, CFD simulations were conducted under pulsatile flow conditions. Aneurysm enlargement significantly altered local flow patterns: an increase in aneurysm size leads to a decrease in flow velocity and a progressively stronger presence of flow recirculation, particularly during the deceleration phase. Enlarged aneurysms consistently displayed lower wall shear stress (WSS) across the aneurysm wall. Oscillatory shear index (OSI) was elevated within the aneurysm regions, with the highest level in the 55 mm and 65 mm models.

These adverse flow characteristics−low WSS, elevated OSI, and distinct flow recirculation−jointly highlight a hemodynamic environment favorable to ILT deposition, localized wall thinning, and increased risk of aortic dissection or rupture. While this study is limited by the assumption of rigid walls and a single patient-specific geometry, this computational framework still offers a practical approach for studying aneurysm progression and rupture risk. Future large-scale studies incorporating FSI models will further enhance our understanding of hemodynamic changes and patient-specific risk assessments for aortic arch aneurysms.

## Figures and Tables

**Table 1:** Mesh sensitivity analysis based on average velocity.

Cell Count (Millions)	Average Velocity (m·s^−1^)	Changes (% Difference)
0.125	0.861	-
0.3	0.747	13.240
1	0.668	10.576
1.91	0.65	2.695

**Table 2:** Timestep size analysis based on average velocity.

Timesteps Size (s)	Average Velocity (m·s^−1^)	Changes (% Difference)
0.01	0.65	-
0.005	0.668	2.769
0.0025	0.667	0.150

**Table 3:** Pressure loss coefficient for different sizes of aneurysm.

	Acceleration	Peak Systole	Deceleration
Healthy	51.929	6.58	−16.32
45 mm	47.58	6.36	−14.19
55 mm	44.89	5.9	−13.09
65 mm	42.08	5.6	−12.13

## Data Availability

The original contributions presented in this study are included in this article. Further inquiries can be directed to the corresponding author upon reasonable request.

## Abbreviations

The following abbreviations are used in this manuscript:

CFD	Computational Fluid Dynamics
WSS	Wall Shear Stress
OSI	Oscillatory Shear Index
TAA	Abdominal Aortic Aneurysm
TKE	Turbulent Kinetic Energy
ILT	Intraluminal Thrombus
FSI	Fluid–structure Interaction
PIV	Particle Image Velocimetry
